# Accumulation of Innate Amyloid Beta Peptide in Glioblastoma Tumors

**DOI:** 10.3390/ijms20102482

**Published:** 2019-05-20

**Authors:** Lilia Y. Kucheryavykh, Jescelica Ortiz-Rivera, Yuriy V. Kucheryavykh, Astrid Zayas-Santiago, Amanda Diaz-Garcia, Mikhail Y. Inyushin

**Affiliations:** 1 Department of Biochemistry, School of Medicine, Universidad Central del Caribe, PO Box 60327, Bayamon, PR 00960-6032, USA; lilia.kucheryavykh@uccaribe.edu (L.Y.K.); 416jortiz@uccaribe.edu (J.O.-R.); yuriy.kucheryavykh@uccaribe.edu (Y.V.K.); 2 Department of Physiology, School of Medicine, Universidad Central del Caribe, PO Box 60327, Bayamon, PR 00960-6032, USA; astrid.zayas@uccaribe.edu (A.Z.-S.); 417adiaz@uccaribe.edu (A.D.-G.)

**Keywords:** amyloid, Aβ peptide, glioma, platelets

## Abstract

Immunostaining with specific antibodies has shown that innate amyloid beta (Aβ) is accumulated naturally in glioma tumors and nearby blood vessels in a mouse model of glioma. In immunofluorescence images, Aβ peptide coincides with glioma cells, and enzyme-linked immunosorbent assay (ELISA) have shown that Aβ peptide is enriched in the membrane protein fraction of tumor cells. ELISAs have also confirmed that the Aβ(1–40) peptide is enriched in glioma tumor areas relative to healthy brain areas. Thioflavin staining revealed that at least some amyloid is present in glioma tumors in aggregated forms. We may suggest that the presence of aggregated amyloid in glioma tumors together with the presence of Aβ immunofluorescence coinciding with glioma cells and the nearby vasculature imply that the source of Aβ peptides in glioma can be systemic Aβ from blood vessels, but this question remains unresolved and needs additional studies.

## 1. Introduction

As Alzheimer’s disease (AD) affects mostly the elderly population [1], gliablastoma (GBM) is the most common primary malignant brain tumor in older people [2]. Recently, statistically independent cohort studies have found an inverse association between cancers in general and AD [3,4,5]. Specifically, most patients with AD are protected from lung cancers [3], and, vice versa, cancer survivors have a lower risk of AD [6]. However, there is a significant positive correlation between the AD mortality rate and the malignant brain tumor mortality rate [4,7,8]. These correlations suggest that there are common factors in these diseases. Mitochondrial metabolism, in general, and the p53, Pin1, and Wnt cellular signaling pathways, in particular, were proposed as possible linkages in this cancer–AD relationship [9,10]. Interestingly, chemotherapy [6] and radiotherapy [9] also affected this correlation. 

On the other hand, the buildup of amyloid precursor protein (APP), the precursor of the AD hallmark amyloid beta (Aβ) peptides, have now been found in pancreatic and breast cancer tumors and the corresponding metastatic lymph nodes [11,12]. Proteolytic cleavage of APP by the α-secretase pathway mediates proliferation and migration in breast cancer, while other pathways were not studied [13]. It was also discovered that plasma levels of Aβ peptides in esophageal cancer, colorectal cancer, hepatic cancer, and lung cancer patients were significantly higher than in normal controls [14]. The question arises, what is the source of these Aβ peptides? Moreover, what is their role?

Aβ peptides can be generated by glioma cells themselves. It was shown that glioma cells in culture produce the 4-kDa Aβ peptide, which co-migrates with synthetic Aβ(1–40) (also known as Aβ40) and is specifically recognized by antibodies raised against terminal domains of the Aβ peptide, and releases them into the medium [15]. The role of Aβ peptides in glioma development was investigated in another study [16]. It was reported that full-length Aβ40 is a dose-dependent inhibitor of angiogenesis and suppresses human U87 glioblastoma subcutaneous xenografts in nude mice. A small peptide sequence of Aβ, Aβ(11–20), was found to be a potent, anti-angiogenic molecule. Systemic delivery of this peptide leads to reductions in glioma proliferation, angiogenesis, and invasiveness [16]. Furthermore, parallel experiments in transgenic mice overexpressing Aβ40 also showed reductions in glioma growth, invasion, and angiogenesis [16,17,18]. 

However, besides glioma itself, there is another systemic source of Aβ peptide production in the body [19,20]. Recently, we showed that platelets produce a massive release of Aβ after thrombosis in the brain and skin and that this release is concentrated near blood vessels [21,22]. It has been shown that platelets are hyperactivated in cancer patients and form cancer cell-induced aggregates and micro-thrombi in the vasculature near tumors (reviewed in [23]). A high platelet count is associated with poor survival in a large variety of cancers, while thrombocytopenia or antiplatelet drugs can reduce the short-term risk of cancer, cancer mortality, and metastasis (reviewed in [24]). Platelets affect glioma cells by releasing platelet-derived growth factor (PDGF) [25]. May platelet-generated Aβ also diffuse to glioma cells and accumulate inside these brain tumors?

In our study, we chose specific antibodies against Aβ peptides with low reactivity for the precursor APP to see whether Aβ immunoreactivity is present in glioma tumors and nearby blood vessels in mice. We used an enzyme-linked immunosorbent assay (ELISA) to study Aβ40 content in tumor and “healthy” brain area, while also assessing Aβ40 content in the membrane and cytoplasmic fractions of glioma cells. The presence of aggregated forms of amyloid inside glioma tumors was evaluated as well. 

## 2. Results

### 2.1. Immunoreactivity against Aβ Peptides Is Present in Glioma Cells in Primary and Secondary Tumors as Well as in Blood Vessels and Erythrocytes in the Near Vicinity, Indicating that the Aβ Level Is Elevated in the Tumor Zone

**Figure 1:**
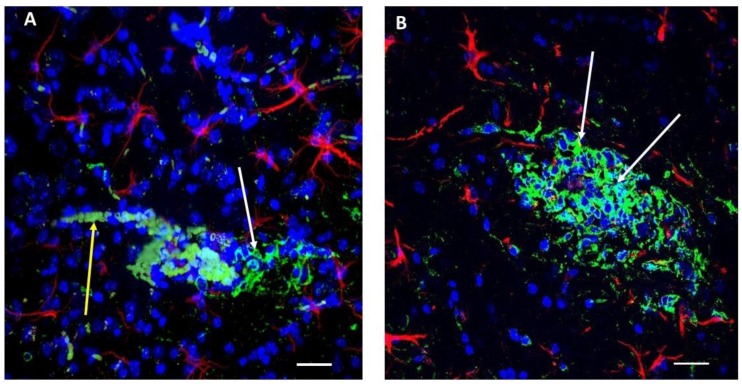
Aβ peptide immunoreactivity (green) in glioma cells and in nearby blood vessels. (**A**) A small glioma tumor near a broken blood vessel. Aβ peptide immunoreactivity (green) visible in glioma cells (white arrow) and in blood vessels. Erythrocytes released from the broken vessel are also marked with Aβ-related immunofluorescence (yellow arrow). (**B**) A larger glioma tumor in which a broken blood vessel passes through the tumor (more clearly visible in the 3D image of this tumor shown in Figure S1B), and white arrows indicate glioma cells marked by green immunofluorescence representing Aβ peptide. For **A** and **B**, astrocytes are indicated by immunoreactivity to GFAP (red) and cell nuclei by DAPI staining (blue). Scale bar, 20 µm. (See also supplemental confocal 3D images of the same tumors in Figure S1A,B, respectively).

After glioma implantation into mouse brains using standard methods established in our laboratory [26,27] we allowed 16 days of tumor growth. We then prepared brain slices containing tumors within nearby tissue. Immunostaining with polyclonal (Figure 1A,B, green) antibody against Aβ showed that these peptides are present in glioma cells (white arrows), in nearby broken blood vessels, and in escaped erythrocytes. In addition, astrocytes are marked by red fluorescence (anti-Glial Fibrillary Acidic Protein (anti-GFAP)), and the nuclei are marked blue (4′,6-diamidino-2-phenylindole (DAPI) staining). The same images (Figure 1A,B) are presented as moving confocal images (Figure S1A,B) so that blood vessel details and their relation to glioma cells are more discernable. Inside blood vessel segments marked by Aβ green immunofluorescence, erythrocytes were also specifically marked by Aβ (Figure 1A,B, see also Figure S1A,B) as well as erythrocytes diffused locally near broken blood vessels (Figure S1A,B), as blood vessels near the tumor are usually ruptured [28]. As was shown previously, Aβ peptide in blood plasma binds to practically all erythrocytes and may be a marker for AD [29]. Also, the addition of synthetic Aβ specifically marks erythrocyte membranes [30]. We want to stress once again that Aβ immunofluorescence is present only in blood vessel segments near the glioma tumor and in the tumor itself (Figure 1A,B and Figure S1A,B). Therefore, only the glioma cells in the tumor and nearby blood vessels containing erythrocytes and within the distance 0–200 µm from the ruptured blood vessel are fluorescent. 

**Figure 2:**
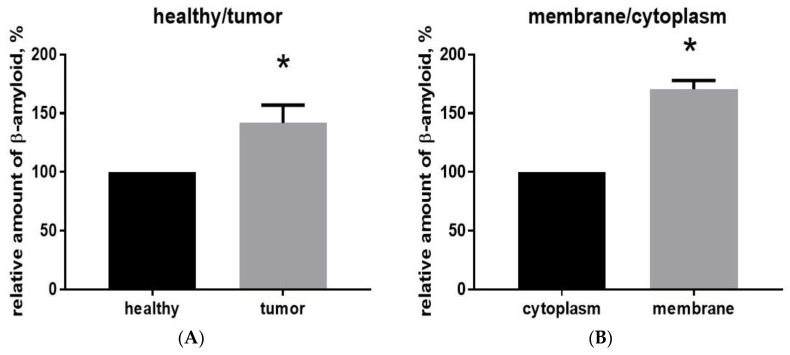
(**A**) The relative amount of Aβ40 in the glioma tissue is elevated. (**B**) Aβ40 in glioma tumor tissue is concentrated in the cell membrane fraction.

We also made ELISA measurements of mouse Aβ40 peptide in the brain sample tissue containing the main tumor versus the “healthy” control from the corresponding cortical zone in the other hemisphere from the same animal 16 days after glioma implantation. Similar amounts of the homogenate were taken for analysis. It was found that the relative amount of Aβ40 in the glioma tissue was 142 ± 9% larger and statistically different (*p* < 0.001; *t* = 4.714; d*f* = 4; *n* = 3) from “healthy” tissue (Figure 2A).

In these experiments, we found that glioma cells exhibit specific Aβ immunofluorescence that clearly marks these cells, but the question arises whether it is inside the cells or somehow attached to the external membrane. 

### 2.2. Aβ40 Is Concentrated in the Membrane Cell Fraction in Glioma Tumor Tissue

To determine more precisely there Aβ is distributed, we separated the cytoplasmic and membrane fractions of proteins from glioma cells from the main tumor extracted from the brain of animals 16 days after implantation. Before processing, blood cells were eliminated from the tumor tissue samples using the Percoll purification method. Membrane and cytoplasmic proteins were isolated, and the total protein content was determined using the Bradford spectrophotometric method to establish a reference point for measuring the amount of Aβ in each fraction. Using ELISA, it was found that the relative amount of Aβ40 in the membrane fraction is significantly greater (170 ± 4%, *p* < 0.001, *t* = 16.23, d*f* = 4, *n* = 3) than in the cytoplasmic fraction (Figure 2B).

### 2.3. Glioma Tumor Tissue Contains Aggregated Amyloid

**Figure 3:**
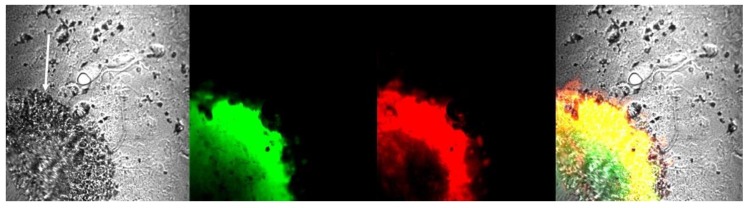
Aggregated amyloid visualized by staining with thioflavin T (green) and thioflavin S (red) inside the glioma tumor body. The white arrow shows the glioma tumor body visible in the brain slice.

To determine whether glioma tumors have aggregated forms of Aβ with cross-β architecture, we used standard thioflavin T and thioflavin S staining of brain slices with glioma from animals with implanted glioma cells. It was previously demonstrated that both thioflavin T and thioflavin S fluorescence originates mainly from dye bound to aggregated forms of amyloids with cross-β-pleated sheet structure, and gives a distinct increase (and a spectral shift in the case of thioflavin T) in fluorescence emission after binding [31,32]. We used IP injection of thioflavin T, while slices containing tumors were additionally stained with thioflavin S. Both dyes specifically marked glioma tumors (Figure 3), in which staining (green for thioflavin T and red for thioflavin S) is obvious only inside the tumor body, while the nearby normal tissue remained unstained. 

## 3. Discussion

Here we report that antibodies against Aβ with relatively low reactivity against APP [33] show Aβ immunostaining in glioma cells and nearby blood vessels in mice (Figure 1). Using ELISA, we also report that Aβ40 levels are significantly increased in glioma (Figure 2). Glioma tissue from one brain hemisphere contains about two-fold more Aβ than a similar amount of tissue from the “mirror” hemisphere, with Aβ concentrated in the membrane fraction. The question arises whether Aβ is coming from the systemic source—from the blood, and is marking the glioma cell membrane—or is synthetized by glioma cells themselves.

Previous studies support the possibility of systemic source for this Aβ. The results indicating increased Aβ content in blood plasma for different types of cancer have already been reported [14]. Systemic Aβ is generated in large quantities by blood platelets in broken vessels, as we have shown for the thrombotic process [21,22]. Here, broken blood vessels marked by extensive Aβ fluorescence can be seen near tumors in our experiments (Figure 1A,B and Figure S1A,B). It has been shown previously that platelets are hyperactivated in cancer patients and form cancer cell-induced aggregates and micro-thrombi in vasculature near tumors (reviewed in [23]), thus suggesting that the source of Aβ that we have found for the clotting process may also be present here. It seems possible that Aβ released from clots can migrate and somehow mark only glioma cells (Figure 1A,B), but this raises new questions about why Aβ marks glioma cells so specifically.

To bind specifically, Aβ must be recognized by a specific receptor on the external membrane of the glioma cell. A known specific Aβ receptor, such as the PrPC–mGluR5 complex, is associated with proline-rich tyrosine kinase 2 (Pyk2 or PTK2B) [34,35]. This receptor localizes to postsynaptic sites in the brain, but is also overexpressed in all glioblastoma cells, where it controls cell migration [27,36]. Aβ is a known inhibitor of Pyk2 [35]. Thus, its release by platelets may be a part of the intrinsic immunity that is directed against cancerous gliomas. Another suspected molecule related to Aβ binding is PI3K (phosphatidylinositol [PI] type 3 receptor tyrosine kinase). This kinase and its signaling network is also present and hyperactivated in a majority of glioblastoma cells, where it controls membrane microdynamics and cell cycling [37,38]. Its Aβ receptor is unknown, but it complexes with PI3K and most probably is situated on the external membrane [39,40]. It is known that Aβ inhibits PI3K activity as well [41]. We speculate that in this case, Aβ peptides generated by platelets also play a role in the intrinsic immunity directed against cancerous gliomas. 

In addition, Aβ may bind to the advanced glycation end products (RAGE) receptor. It is known that this receptor is the binding site for Aβ peptides [42] thus mediating Aβ transport through the blood–brain barrier [43]. Very same RAGE receptor regulates the tumor environment and tumor cell migration, is part of the important microglial activation mechanism and is overexpressed in tumors [44].

On the other hand, it was shown that glioma cells in culture produce Aβ peptides that comigrate with synthetic Aβ40 and are specifically recognized by antibodies raised against the terminal domains of the Aβ peptide and released by these cells into the medium [15]. However, cultured and in vivo astrocytes also produce Aβ peptides is similar amounts [45,46,47] and astrocytes were not marked by Aβ immunofluorescence in our experiments, probably because these peptides is present in/near the astrocytes in amounts that can be neglected compared with the glioma tumor cells that we have studied here. While derived from the same cell type, glioma cells are clearly marked by Aβ immunofluorescence in our experiments (Figure 1).

It is clear that the question of whether the source of Aβ is inside the glioma cell itself or is a systemic source from blood vessels should be investigated further. Anyway, all our results from these experiments taken together as well as our previous experience with Aβ peptides released during platelet accumulation and aggregation in thrombotic blood vessels [21,22] lead us to the conclusion that most probably Aβ peptides are generated by platelets and somehow bind almost exclusively to glioma cells. 

An additional issue is the accuracy of Aβ40 concentrations measurements in brain tissue. In our study of Aβ40 concentrations in tissue, we used relative values, indicating the percentage change from initial values, as the most accurate. It was shown previously that the Invitrogen Aβ40 ELISA Kit is very specific to murine Aβ40, but the data are very sensitive to “noise” (such as the presence of other proteins and lipids), and absolute values can deviate 40–50% [48]. Also, ELISA data may vary considerably, with a variety of collection and storage protocols [49]. Measurement of Aβ by ELISA reveals mainly free peptides, while a significant amount of Aβ peptide remains bound to proteins, lipoproteins, and cell membranes [50].

Our experiments also indicate that there is some thioflavin-positive amyloid inside glioma tumors (Figure 3). While we have shown that Aβ peptides are definitely present in tumor and may constitute a predominant part of this glioma amyloid, the specific type of aggregated amyloid found inside the borders of glioma tumors is unknown. To our opinion, this amyloid is most probably mixed amyloid, as was found for AD [51]. Protein aggregation is sequence specific, not favoring self-assembly over cross-seeding with nonhomologous sequences [52]. However, proteins with aggregation-prone regions may aggregate with each other at elevated concentrations, forming a mixed misfolded amyloid [53]. In this case, one aggregated protein can work as a “seed” for aggregation of other protein types. Previously, different amyloids were found in a variety of tumors. Different carcinomas have amyloid stroma [54,55], and odontogenic tumors are positive for thioflavin T and Congo Red staining and are also immunopositive for the enamel matrix protein ameloblastin [56,57,58]. Similarly, amyloid was reported in breast cancer tumors but was determined to be a localized amyloid light chain (AL) type (primary amyloidosis caused by ImG light-chain β-sheeting) [59,60]. Localized AL type amyloidosis was also found in myeloma (plasma cell) tumors as well as in kidneys and early-stage non-small-cell lung adenocarcinomas [61]. If the content of amyloid in glioma tumors is mixed, it must be further studied, because tumor-related amyloid could be a new target for anticancer therapy. 

## 4. Materials and Methods 

### 4.1. Ethics Statement

All procedures involving rodents were conducted in accordance with the National Institutes of Health regulations concerning the use and care of experimental animals and approved by the Universidad Central del Caribe Institutional Animal Care and Use Committee. All efforts were made to minimize suffering. In all surgical experiments, animals were anesthetized with isoflurane (4% for induction and 1.75% for maintenance) using a Matrix Quanti-flex VMC Anesthesia Machine for small animals (Midmark Corporation, Dayton, OH, USA). The animals were sacrificed for brain tissue and blood analysis after experiments.

### 4.2. Glioma Cell Culture

The GL261 glioma cell line derived from C57BL/6 mice was obtained from the NCI (Frederick, MD, USA). All cells were cultured in Dulbecco’s Modified Eagle’s Medium (DMEM) supplemented with 10% fetal calf serum, 0.2 mM glutamine, and antibiotics (50 U/mL penicillin, 50 μG/mL streptomycin) and maintained in a humidified atmosphere of CO_2_/air (5%/95%) at 37 °C. The medium was exchanged with fresh culture medium every 2–3 days.

### 4.3. Intracranial Implantation of Glioma Cells

All surgery was performed under isoflurane anesthesia, and all efforts were made to minimize suffering. GL261 glioma cells were implanted into the right cerebral hemisphere of 12–16-week-old C57BL/6 mice. Implantation was performed according to the protocol that we described earlier [26]. Briefly, mice were anesthetized with isoflurane, and a midline incision was made on the scalp. At stereotaxic coordinates of bregma, 2 mm lateral, 1 mm caudal, and 3 mm ventral, a small burr hole (0.5 mm diameter) was drilled into the skull. One microliter of cell suspension (2 × 10^4^ cells/μL in phosphate buffer solution (PBS)) was delivered at a depth of 3 mm over 2 min. Sixteen days following injection, the animals were anesthetized with pentobarbital (50 mg/kg) and transcardially perfused with PBS followed by 4% paraformaldehyde (PFA). The brains were removed and post-fixed in 4% PFA/PBS for 24 h at 4 °C, followed by 0.15 M, 0.5 M, and 0.8 M sucrose at 4 °C until fully dehydrated. The brains were then frozen and embedded in Cryo-M-Bed embedding compound (Bright Instrument, Huntingdon, UK) and cut using a Vibratome UltraPro 5000 cryostat (American Instrument, Haverhill, MA, USA). 

### 4.4. Percoll Purification of Blood Cells from Tissue Samples for Membrane Fraction Isolation

To study Aβ distribution inside tumor cells, we first eliminated blood cells from the tumor tissue sample using the Percoll purification method. Tumors and healthy cortex from the contralateral hemisphere were removed from the mouse brains, minced into 1–2-mm pieces with a razor blade, and enzymatically homogenized using a collagenase/hyaluronidase in DMEM (cat. #07912, Stemcell Technologies, WA, USA). Blood cells were separated from the homogenized tissue using Percoll (Sigma-Aldrich, St. Louis, MO, USA) gradients of 30% and 70%. Following this procedure the tissue fraction free from blood cells was collected from the top of the 70% Percoll level and used for further analysis.

### 4.5. Isolation of Membrane and Cytoplasmic Proteins

A homogenized cell suspension was resuspended and sonicated in 20 mM Tris buffer containing 1 mM ethylenedinitrilotetraacetic acid (EDTA), 1 mM β-mercaptoethanol, and 5% glycerin, pH 8.5 with HCl, 1 µM Na_3_VO_4_, 0.5 mM phenylmethylsulfonyl fluoride (PMSF), and 10 mM dithiothreitol (DTT). After centrifugation the supernatant was collected and used for further investigations as the cytoplasmic protein fraction. The pellet containing the membranes and the membrane proteins was lysed, and clarified cell lysate was used as the membrane protein fraction. 

### 4.6. Enzyme-Linked Immunosorbent Assay (ELISA) Measurements

A specialized, ready-to-use, mouse-specific, solid-phase sandwich ELISA kit (cat. #KMB3481; Invitrogen, Thermo Fisher Scientific, Waltham, MA, USA) was used for direct measurement of the amount of Aβ40 peptide in the brains of experimental animals in accordance with the manufacturer’s documentation. Briefly, the brain samples were homogenized mechanically, and 100 mg of homogenate was then lysed in guanidine solution (5 M guanidine HCl, 50 mM Tris HCl, pH 8.0). In other experiments, the lysate (normalized to total protein content) from membrane and cytoplasmic fractions (see above) were used. A monoclonal antibody against the NH_2_-terminus of mouse Aβ40 peptide was coated onto the wells of the microtiter strips provided in the kit. Samples, including standards of known Aβ40 content for calibration purposes as well as experimental specimens, were pipetted into the wells. After washing, the rabbit antibody specific to the COOH-terminus of Aβ40 was added and detected with horseradish peroxidase-labeled anti-rabbit antibody. The optical density values at 450 nm were determined using a Wallac 1420 Victor 2 Microplate Reader (PerkinElmer Inc., Waltham, MA, USA). The calculated mean reading from the healthy hemisphere (normalized cytoplasmic fraction) was defined as 100%, while other readings were presented as the percentage of this value. 

### 4.7. Immunohistochemistry and Confocal Microscopy

Immunostaining was performed using a protocol previously established in our laboratory [22,62]. Frozen 30-µm sections were generated from brain cortex containing the tumor(s). The sections were blocked with 5% normal goat serum/5% normal horse serum (Vector Laboratories, Burlingame, CA, USA) in 0.10 M phosphate buffer solution (PBS: NaCl, 137 mM; KCl, 2.70 mM; Na_2_HPO_4_, 10.14 mM; KH_2_PO_4_, 1.77 mM) containing 0.3% Triton X-100 and 0.05% phenylhydrazine for 60 min for permeabilization and then processed separately using two different antibodies against Aβ. For that purpose, slices were incubated with a rabbit polyclonal antibody to Aβ (Abcam, Cambridge, MA, USA, cat. #ab2539) diluted 1:400 in 0.03% Triton X-100, 1% dimethyl sulfoxide (DMSO), 2% bovine serum albumin (BSA), 5% normal horse serum, and 5% normal goat serum in 0.1 M PBS. Anti-GFAP–Cy3 (1:200) was added, and the slice left overnight at 4 °C. After three washes with permeabilization solution for 10 min, the secondary antibodies (fluorescein-labeled goat anti-rabbit IgG) were added at a dilution of 1:200 with shaking for 2 h at room temperature and protected from light. The slices were then washed three times with PBS for 10 min and once with distilled water before being transferred onto a glass slide containing Fluoroshield mounting medium (Sigma-Aldrich, St. Louis, MO, USA, cat. #F6057) with DAPI. Negative controls were routinely performed by removal of primary antibody in each staining experiment to validate the immunohistochemical staining quality and results. 

For thioflavin (Th) staining we used: (1) ThT staining, in which mice were injected IP with 10 µL/g of 3 mM solution of ThT in PBS. After 5 min, the animals were euthanized, and the brains were harvested and kept in fixative without light. (2) ThS staining, in which brain slices (30 µm) containing tumors were allowed to completely air dry prior to staining, then stained with a drop of 3 mM ThS in PBS (previously filtered through a 0.2-μm filter) for 5 min, then washed twice with distilled water and dried again. The coverslip was mounted with a drop of Vaseline on the slice. DAPI and Cy3 excitation/emission filters were used to visualize ThT and ThS fluorescence, respectively. 

Images were acquired using an Olympus Fluoview FV1000 scanning inverted confocal microscope system equipped with a 20×, 40×, or 60×/1.43 oil objective (Olympus, Melville, NY, USA). The images were analyzed using ImageJ software (http://imagej.nih.gov/ij) with the Open Microscopy Environment Bio-Formats library and plugin, allowing for the opening of Olympus files (http://www.openmicroscopy.org/site/support/bio-formats5.4/). The data were evaluated using custom colorization.

### 4.8. Statistics and Measurements

Using GraphPad Prism 7.03 (GraphPad Software, Inc., La Jolla, CA, USA) for calculations, an unpaired *t*-test was employed to estimate statistical differences. Values were determined to be significantly different if the two-tailed *p*-value was <0.05.

## 5. Conclusions

- Aggregated amyloid is present inside glioma tumor borders;
- Aβ peptide immunofluorescence is present in glioma tumors, marking glioma cells and nearby ruptured blood vessels.

## Supplementary Materials

Supplementary materials can be found at https://www.mdpi.com/1422-0067/20/10/2482/s1.

## Abbreviations

ELISA	enzyme-linked immunosorbent assay
ThS	Thioflavin S
ThT	Thioflavin T
PMSF	Phenylmethylsulfonyl fluoride
DTT	Dithiothreitol
DMEM	Dulbecco’s Modified Eagle’s Medium
Aβ	Amyloid beta peptide
