# Supplementary figures and images for: Accumulation of Innate Amyloid Beta Peptide in Glioblastoma Tumors
Source: Int J Mol Sci. 2019 May 20;20(10):2482. doi: 10.3390/ijms20102482 (PMC6567111; doi:10.3390/ijms20102482)

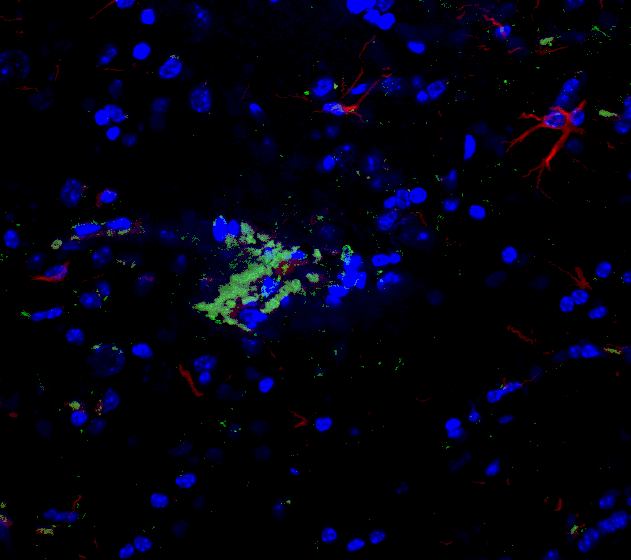

Supplement: Supplementary file 1 [file ijms-20-02482-s001.zip › ijms-491598-SI final/Fig S1A gif.gif]

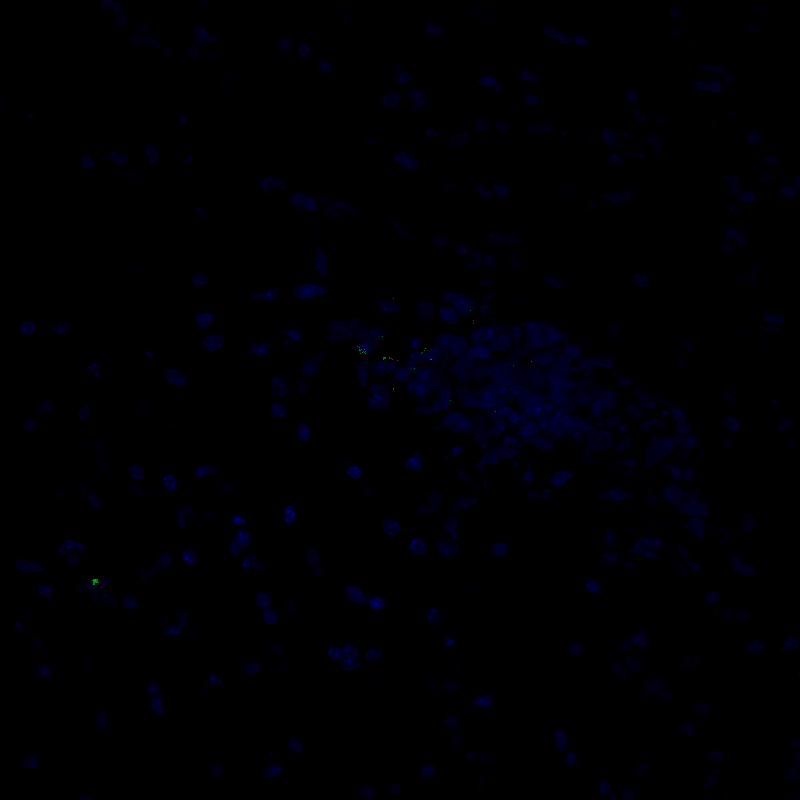

Supplement: Supplementary file 1 [file ijms-20-02482-s001.zip › ijms-491598-SI final/Fig S1B gif film.gif]
